# Phylogenetic studies reveal existence of multiple lineages of a single genotype of DENV-1 (genotype III) in India during 1956–2007

**DOI:** 10.1186/1743-422X-6-1

**Published:** 2009-01-06

**Authors:** Himani Kukreti, Paban Kumar Dash, Manmohan Parida, Artee Chaudhary, Parag Saxena, RS Rautela, Veena Mittal, Mala Chhabra, D Bhattacharya, Shiv Lal, PV Lakshmana Rao, Arvind Rai

**Affiliations:** 1 Division of Biochemistry and Biotechnology, National Institute of Communicable Diseases (NICD), 22 Shamnath Marg, Delhi 110054, India; 2 Division of Virology, Defence Research & Development Establishment, Jhanshi Road, Gwalior-474002, MP, India; 3 Division of Zoonosis, National Institute of Communicable Diseases (NICD), 22 Shamnath Marg, Delhi 110054, India

## Abstract

**Background:**

Dengue virus type 1 (DENV-1) have been mostly circulating silently with dominant serotypes DENV-2 and DENV-3 in India. However recent times have marked an increase in DENV-1 circulation in yearly outbreaks. Many studies have not been carried out on this virus type, leaving a lacunae pertaining to the circulating genotypes, since its earliest report in India. In the present study, we sequenced CprM gene junction of 13 DENV-1 isolated from Delhi and Gwalior (North India) between 2001–2007 and one 1956 Vellore isolate as reference. For comparison, we retrieved 11 other Indian and 70 global reference sequences from NCBI database, making sure that Indian and global isolates from all decades are available for comparative analysis.

**Results:**

The region was found to be AT rich with no insertion or deletion. Majority of the nucleotide substitutions were silent, except 3 non-conservative amino acid changes (I → T, A → T and L → S at amino acid positions 59,114 and 155 respectively) in the Indian DENV-1 sequences, sequenced in this study. Except two 1997–98 Delhi isolates, which group in genotype I; all other Indian isolates group in genotype III. All Indian genotype III DENV-1 exhibited diversity among them, giving rise to at least 4 distinct lineages (India 1–4) showing proximity to isolates from diverse geographic locations.

**Conclusion:**

The extensive phylogenetic analysis revealed consistent existence of multiple lineages of DENV-1 genotype III during the last 5 decades in India.

## Background

Dengue fever (DF) is one of the most important arboviral diseases of humans in tropic and sub-tropics [1,2]. In South-East Asia, with a total population of 1.5 billion, approximately 1.3 billion people live at risk of acquiring DF or DHF [3,4]. Its etiological agent, Dengue virus belongs to family *Flaviviridae*, genus *Flavivirus*; and exists in 4 antigenically distinct serotypes, Dengue virus type 1–4 (DENV-1 to 4) [5]. Although history of dengue virus in India dates back to 1946, however the first major outbreak was reported in 1963 in Calcutta. Since then many outbreaks have been reported from all over the country [6-9]. Although all 4 dengue serotypes have been reported to circulate in the country [10] but only DENV-2 and DENV-3 have been implicated in major DF/dengue hemorrhagic fever (DHF) outbreaks [11-14]. We and some other workers have earlier reported CprM gene based genotyping of DENV-2 and DENV-3 which has proved to be useful for carrying out molecular epidemiology of these viruses [12,14-16]. Lately there has been a rise in DENV-1 associated cases, which account for around 30% of the total cases in most recent 2006 DF outbreak, when it co-existed with pre-dominant DENV-3 [17]. Earlier DENV-1 has been reported from south India (Vellore) in 1956 and 1962–64; and from north India (Delhi and Gwalior) in 1970, 1982, 1997–98 and 2002–2006. Inspite of all these reports, no attempt has been made to study the phylogeny of this virus since its emergence. With increase in DENV-1 cases, the need to understand the genetic nature of circulating DENV-1 has become even more necessary as it will harbors significant information regarding the genotypes of the DENV-1 circulating in the country for so long. In addition it will also indicate whether these viruses were pre-existing or an importation. With this view, present study was undertaken to understand the genetic nature of circulating DENV-1 in India and to trace their evolution during the last 50 years, by sequencing CprM gene junction of 13 DEN-1 isolated in India during 2001–2007 and comparing 354 bp of this region with 11 other Indian DENV-1 sequences reported till date with at least one sequence from each decade, since it was first reported in the country. Seventy other global reference sequences were also retrieved from NCBI nucleotide database for comparison. This study shall help to fill the lacunae pertaining to knowledge of circulating DENV-1 genotypes in India since its first report.

## Results

**Figure 1:**
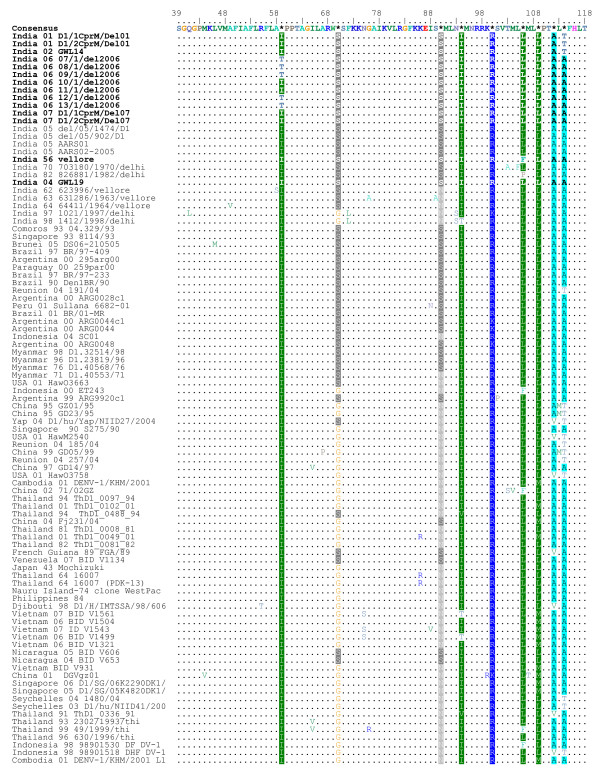
**Amino acid alignment of CprM gene junction sequences of all Indian and global DENV-1 showing changes in comparison to the consensus sequence**. The Indian sequences that are sequenced in this study are in bold. The numbering of amino acid position corresponds to the ORF of DEN-1 strain "Singapore 8114/93" (GenBank Acc. No. AY762084). Dot (.) indicates amino acid similarities with the consensus.

**Figure 2:**
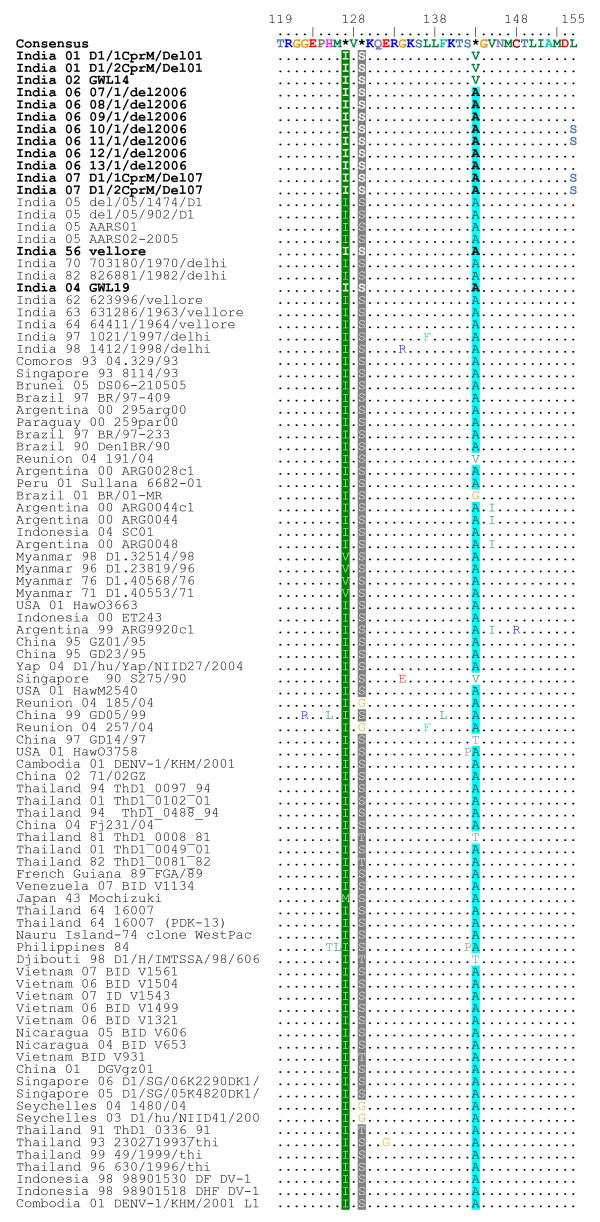
**Amino acid alignment of CprM gene junction sequences of all Indian and global DENV-1 showing changes in comparison to the consensus sequence**. The Indian sequences that are sequenced in this study are in bold. The numbering of amino acid position corresponds to the ORF of DEN-1 strain "Singapore 8114/93" (GenBank Acc. No. AY762084). Dot (.) indicates amino acid similarities with the consensus.

Thirteen serum samples found positive for DENV-1 during dengue outbreaks in northern India during 2001–2007 were included in this study. For sequence comparison and phylogenetic analysis we selected a 354 bp sequence (nucleotides 208–561) from CprM gene junction of 13 DENV-1 sequenced in this study and compared them with 11 representatives Indian and 70 other global geographically diverse DENV-1 sequences, spanning last 5 decades. All these sequences were aligned with the prototype Indian DENV-1 isolate (India-56 (Vellore) that was also sequenced in the study. This region was found to be AT rich and the AT composition of the Indian DENV-1 varied from 52.6–53.7%. The alignment did not reveal any base insertion or deletion, only substitutions which were mostly synonymous in nature. Deduced amino acid alignment of the Indian isolates sequenced in this study revealed that the non-synonymous nucleotide substitutions, gave rise to only 3 non-conservative amino acid changes i.e. isoleucine to threonine (Amino acid position 59) and leucine to serine (Amino acid position 155) as seen in some 2006 and 2007 Delhi isolates; and alanine to threonine (Amino acid position 114) as observed in 2001 Delhi and 2002 Gwalior isolates (amino acid alignment shown as Figure 1 and 2).

On comparison of sequences, we observed that one 2002 Gwalior and two 2001 Delhi CprM sequences exhibited close nucleotide identity of 99.7% to a 2004 Reunion Island sequence. It was also found that the most recent Indian isolates from 2006 and 2007 show mean sequence identities of 96.45%, 95.35%, 97.5% and 96.75% with 1956 Vellore, 1970 Delhi, 1982 Delhi and 2004 Gwalior sequences; and 98.6–99.2% (mean 98.9%) nucleotide identity among themselves. On analyzing the Indian reference sequences taken for comparison, it was observed that 2005 Delhi sequences exhibited 95.8–99.2% (mean 97.5%) identity with sequences from South American isolates sampled between 1989–2001. Three sequences from south India (Vellore) sampled in 1962–64 show a nucleotide identity of 96.6–96.9% (mean 96.75%) with a 1956 sequence from the same area. Two Delhi sequences from years 1997 and 1998 show more nucleotide identity i.e. 95.8–96.6% (mean 96.2%) with Japan Mochizuki sequence isolated in 1943; than to the other Indian sequences, with whom theses two sequences show a nucleotide identity of only 93%.

**Figure 3:**
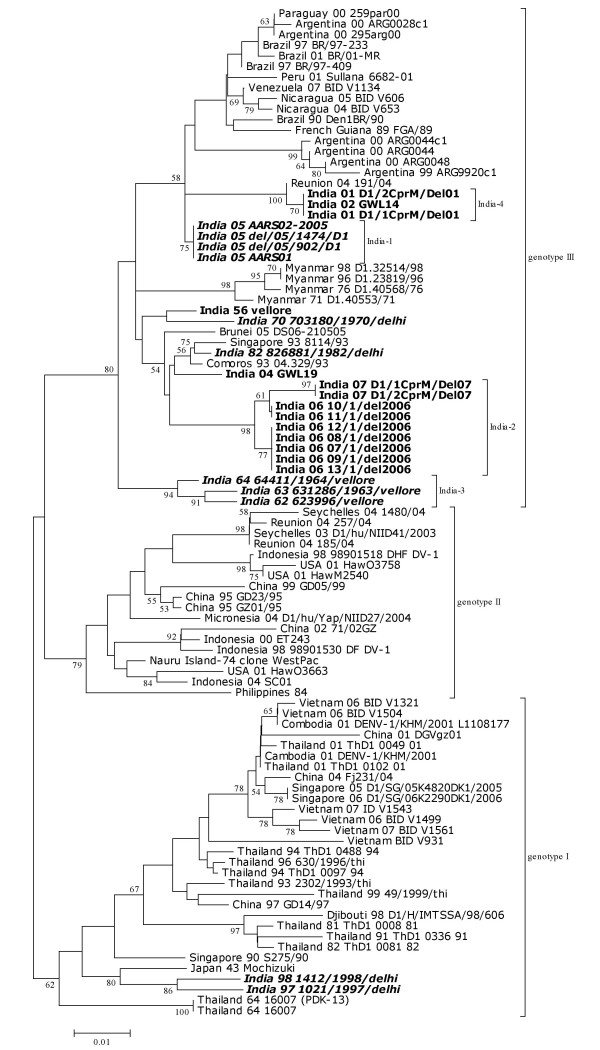
**DENV-1 phylogenetic tree**. Tree was generated by Neighbor-Joining method based on 354 bp nucleotide sequences of CprM gene region. Each isolate is denoted by country of isolation and last two digits of year of isolation, followed by virus ID. Bootstrap support values (>50%) are shown for major nodes on the tree. All horizontal branch lengths are drawn to scale. DENV-1 sequences that were sequenced in the study are in bold and other Indian sequences taken for comparison are in bold italics.

On carrying out the phylogenetic analysis of all these DENV-1, on basis of CprM sequences, we observed that they grouped in 3 distinct clusters (Figure 3) or genotypes with a mean inter-genotype sequence divergence ~7.2%. All the Indian isolates, grouped in genotype III except 2 Delhi 1997 and 1998 isolates which grouped in genotype I showing closeness to 1943 Mochizuki strain from Japan. All the Indian Isolates classified in genotype III show intra-genotypic clustering giving rise to 4 distinct Indian lineages (India-1 through 4) and some Indian isolates although clustering together but not giving rise to a separate lineage. India-1 lineage consists of DENV-1 from Delhi isolated in 2005 and is close to South American viruses isolated from Brail, Argentina, Nicaragua, Paraguay, Peru and Venezuela between 1990–2007. Lying close to this lineage is another lineage, India-4 clustering with a 2004 African isolate from Reunion Island. Delhi 2001 and Gwalior 2002 sequences exhibit 2 C → T (at nucleotide position 274 and 522) and 2 G → A (at nucleotide position 434 and 436) transitions, which they share with 2004 Reunion island sequence. Alanine to threonine amino acid change at position 114 was also observed exclusively in this lineage and hence considered as a lineage specific change (Figure 1). Designation of these Indian lineages i.e. India-1–4 do not signify a chronological order, however they were sequentially designated in order of discovery of these lineages. 1962–64 strains from South India (Vellore) also formed a different lineage (India-3) that diverged long ago, with no other Indian and global isolates clustering in this group. There were other Indian sequences which clustered together but did not form a distinct grouping or lineage. These include 1956 Vellore isolate, 1970 and 1982 Delhi isolates and 2004 Gwalior isolate. These sequences were close to 1993 isolates from Singapore and Comoros and a 2005 isolate form Brunei. All these sequences showed a common T → C transition at nucleotide position 208. These DENV-1 were also close to most recent isolates from Delhi sampled in 2006 and 2007 which clustered together to give rise to another independent lineage (India-2) that exhibited 3 lineage specific nucleotide changes (A → G, C → T and T → G at nucleotide position 307, 469 and 520). Amino acid changes Isoleucine to threonine (at position 59 in Figure 1) and leucine to serine (at position 155 in Figure 2) were also observed in some and not all isolates in this lineage and hence could not be designated as lineage specific changes.

Genotype I in which two of the Indian isolates from Delhi (1997–98) cluster; consists of viruses from Asia except 1 African isolate from Djibouti isolated in 1998. Although, most Asian strains in this genotype are from South East Asian countries viz. Cambodia, Vietnam, Thailand and Singapore; 2 recent isolates from China and 1 very old strain from Japan (Japan 43 Mochizuki strain) are the only 3 strains from North Asia that lie in this genotype.

None of the Indian strains was classified as genotype II. This genotype was more cosmopolitan in nature with strains from East Africa, Asia and South Pacific. East African strains include recent viruses from East African islands of Seychelles and Reunion isolated during 2003–04; South Pacific strains include viruses from Hawaii, Nauru Island and Micronesia isolated between 74-04; and Asian strains include viruses from China, Philippines and Indonesia isolated during 1995–2004. Although African strains are clustering together, making a distinct grouping; strains from South Pacific and Asia do not show any such geographical clustering within this genotype.

## Discussion

The large scale climatic and demographic changes have resulted in distribution of *Aedes *species in hitherto unknown areas. This has led to appearance of dengue infection in many newer areas with an estimated 3 billion people living at risk of this infection around the world. Considering the high rates of *Aedes *infestation and the presence of high-risk susceptible population, the probability of dengue epidemic always remain very high in India [18]. The pathogenesis of dengue is not yet fully understood. Though re-infection with a heterologous dengue serotype remain a major risk factor for DHF, however, occurrence of DHF even in primary infection makes the role of virus in pathogenesis all the more important. The incursion of new genotype into an area is also being attributed to the severe form of the disease [19]. All these facts drive the scientific community to pay more attention towards the genetic nature of dengue viruses and their spread in the population.

Like other RNA viruses, the dengue viruses also revealed strong genetic diversity. Dengue serotypes are further classified into 4–5 'genotypes' based on their genetic diversity [20]. However in recent reports many researchers have categorized DENV-1 in three distinct genotypes [21-23]. Researchers have also reported clustering of sequences below the genotype level that correlate with the geographical origin and/or time of isolation and defined as lineages/clades [21,23,24]. Different regions of dengue genome like Envelope, E-NS1, C-prM and complete genome has been utilized for the genotyping [15,20,21,24]. We have utilized the sequence information of CprM gene junction in this study. The genotyping based on the CprM gene junction has been adopted by several researchers including us in the recent past [12,14,15,25]. This results in faster and economical genotyping due to utilization of a single set of primer pair for both amplification and sequencing [26].

The involvement of DENV-1 in major dengue outbreaks in India was not recorded. Most of the major dengue outbreaks are attributed to DENV-2 and DENV-3 viruses. However, DENV-1 were isolated from different parts of India at regular intervals, since its first isolation from southern India (Vellore) in 1956. It is increasingly implicated as a minor serotype during recent outbreaks in India, including the Delhi outbreak in 2006 [17,27]. The lack of sequence analysis of Indian DENV-1 also affects the effective monitoring of circulating genotypes in India. In this study, we determined the nucleotide sequence of 13 recent DENV-1 directly from clinical samples to avoid selection and sequencing of mutant viral RNA which may occur during isolation/propagation in mouse brain/cell culture [28]. The comparison of deduced amino acid sequence revealed 3 important unique substitutions in 2001–02 and some of the 2006–07 Indian isolates. These involve substitutions of hydrophobic Isoleucine and Leucine by hydrophilic Threonine and Serine respectively in some 2006–07 isolates; and hydrophobic Alanine by hydrophilic Threonine in Delhi 2001 and Gwalior 2002 isolates. The hydrophilic amino acids are found mostly on the surface of the protein and are involved in the immunological interactions. The implications of these unique changes in recent Indian DENV-1 warrants further studies to understand their virulence and epidemic potential.

The phylogenetic analysis revealed the Indian DENV-1 collected over last 5 decades fall into two genotypes (I and III). However, majority of the viruses are grouped within genotype III, two isolates sampled from Delhi during 1997–98 group in genotype I. These genotype I viruses from Delhi were found closely related to the 1943 Japanese Mochizuki strain. Their proximity was however difficult to explain. Similar type of genotype I viruses were also found circulating predominantly in other Asian countries including Thailand and China around the same time. Presence of an African isolate from Djibouti in this group probably signifies the introduction of these Asian viruses in Djibouti or vice-versa. Thailand strains belonging to this genotype were isolated over four decades (1964–2001); indicating persistence of this genotype in Thailand for a long time. However, the restricted circulation of genotype I viruses in Delhi for just 2 years is very surprising.

The phylogenetic analysis clearly revealed continuous circulation of genotype III viruses over the last 50 years in India. Interestingly, majority of the Indian genotype III viruses are found to be phylogenetically quite distinct. It is surprising to find that the recent viruses recovered in quick succession in 2001–02, 2004 and 2006–07 from northern India also belong to separate lineages. In contrast, this type of distinct lineage pattern was not observed among Indian DENV-2 and 3 [12,14].

Domingo et al., [24] has earlier established 2 distinct lineages of DENV-1 in India and designated them as India-1 and India-2. While India-1 has shown closeness to South American strains, India-2 exhibited close proximity to a 1993 isolate from Singapore. In our earlier study we have substantiated the presence of India-1 and India-2 lineages during 2005 and 2006 respectively and also reported a third lineage that was present in south India (Vellore) between 1962–64, whose present existence could not be established [17]. In this study we now report another lineage, which consists of viruses from Delhi and Gwalior sampled in 2001 and 2002 respectively; that we designated as India-4. During the same period presence of India-2 lineage was also reported [24], there by suggesting the co-circulation of India-2 and India-4 lineage in India during 2001. As evident from the phylogenetic tree (Figure 3) Indian DENV-1 isolated in 1956, 1970, 1982 and 2004 could not be designated in a separate lineage but their relatedness could be deciphered by clustering together of these isolates in the phylogenetic tree.

In the cosmopolitan genotype (genotype II) with viruses from East Africa, Asia and South Pacific, only African isolates clustered together indicating independent evolution of these viruses in this area.

## Conclusion

Thus we conclude that during the last 5 decades there has been a persistence of genotype III of DENV-1 in India with genotype I being present only during 2 years i.e. 1997–98 in Delhi. Most of the Indian DENV-1, grouping in genotype III were quite diverse, giving rise to distinct lineages. Thus, there has been persistence of multiple lineages of DENV-1 genotype III during the last 50 years in India.

This study clearly identified the genotypes of DENV-1 circulating in India since 1956. It also confirmed the utility of CprM gene junction for rapid and economical genotyping in endemic areas. The prevalence of multiple lineages of DENV-1 in India warrants sustained monitoring of the circulating viruses, to implement effective control measures at the earliest.

## Materials and methods

### Clinical samples

Serum samples from febrile patients suspected for dengue infection were collected from Delhi and Gwalior during 2001–2007. Informed consent from all the patients and/or their parents (in minors) was obtained, before collection of clinical samples. Approval of the ethical committee of both institutions was obtained to carry out the present study. Thirteen serum samples found positive for DENV-1 RNA were included in this study.

### Virus

Dengue virus serotype 1 (P-23086) obtained from the National Institute of Virology (NIV), Pune, India was used as reference strain in this study. This is the prototype Indian DENV-1 virus isolated from Vellore, India in 1956.

### Extraction of viral RNA

Viral RNA was extracted from 140 μl of serum samples and P-23086 infected C6/36 supernatant by using QIAamp viral RNA mini kit (Qiagen, Germany) in accordance with the manufacturer's instructions. Finally RNA was eluted in 50 μl of nuclease free water and stored at -80°C until use.

### Reverse transcription- Polymerase chain reaction (RT-PCR)

The RT-PCR was carried out in a 25 μl reaction volume using the access quick one-step RT-PCR kit (Promega, USA) containing PCR master mix, AMV-RT, and respective sense and antisense primers [(D1: 5'-TCAATATGCTGAAACGCGCGAGAAACCG-3') (D2: 5'-TTGCACCAACAGTCAATGTCTTCAGGTTC-3')] [26] in a thermal cycler (BioRad, USA). The thermal profile of the RT-PCR reaction was- RT step at 42°C for 45 min, followed by PCR step of initial denaturation at 95°C for 2 min, followed by 35 cycles of denaturation at 95°C for 1 min, annealing at 55°C for 1 min, extension at 72°C for 2 min and final extension at 72°C for 10 min. The PCR products were gel purified from 1.2% agarose gel using the QIAquick PCR purification kit (Qiagen, Germany) and used as template in sequencing reaction.

### Sequencing reaction

Both strands of the purified amplicons were sequenced employing Big dye terminator cycle sequencing ready reaction kit (Applied Biosystems, USA) following the standard protocol described by us earlier [12]. The cycle sequenced product was purified by precipitation with 75% isopropanol and was vaccum dried. The DNA pellet was resuspended in 10 μl of template suppression reagent (TSR), heated at 95°C for 2 min and loaded on the ABI 310 automated DNA sequencer (Applied Biosystems, USA).

### Sequence alignment and phylogenetic analysis

The CprM gene sequences of fourteen Indian DENV-1 obtained in the present study were submitted to GenBank at  (accession numbers are mentioned in Table 1). BLAST search was carried-out to confirm the identity of strains. For comparison, we retrieved 11 DENV-1 CprM sequences from India and 70 belonging to diverse geographical locations from the global data base as shown in phylogenetic tree (Figure 3). Lasergene 5 software package (DNASTAR Inc, USA) was used to examine the percent identity and diversity among sequences. Sequences were translated into amino acid and aligned using BioEdit v7.0.9. Phylogenetic analysis was carried out using MEGA version 3.1 [29]. Phylogenetic tree was constructed employing Neighbor Joining method [30] with bootstrap analysis of 10000 replicates.

**Table 1:** Details of all the Indian DENV-1 isolates included in this study.

**Isolated Viruses**	**Year of Isolation**	**GenBank** **Accession No**.	**Genotype**
**India 56 vellore**	**1956**	EU626489	**genotype III**
India 62 623996/vellore	1962	AY593211	genotype III
India 63 631286/1963/vellore	1963	AY593212	genotype III
India 64 64411/1964/vellore	1964	AY593214	genotype III
India 70 703180/1970/delhi	1970	AY593215	genotype III
India 82 826891/1982/delhi	1982	AY593217	genotype III
India 97 1021/1997/delhi	1997	AY584591	genotype I
India 98 1412/1998/delhi	1998	AY584594	genotype I
**India 01 D1/1CprM/Del01**	**2001**	EU846232	**genotype III**
**India 01 D1/2CprM/Del01**	**2001**	EU846233	**genotype III**
**India 02 GWL14**	**2002**	EU626490	**genotype III**
**India 04 GWL19**	**2004**	EU626491	**genotype III**
India 05 del/05/1474/D1	2005	EF064776	genotype III
India 05 del/05/902/D1	2005	EF064774	genotype III
India 05 AARS01	2005	EF222443	genotype III
India 05 AARS02-2005	2005	EF222444	genotype III
**India 06 07/1/del2006**	**2006**	EU181194	**genotype III**
**India 06 08/1/del2006**	**2006**	EU181195	**genotype III**
**India 06 09/1/del2006**	**2006**	EU181196	**genotype III**
**India 06 10/1/del2006**	**2006**	EU181197	**genotype III**
**India 06 11/1/del2006**	**2006**	EU181198	**genotype III**
**India 06 12/1/del2006**	**2006**	EU181199	**genotype III**
**India 06 13/1/del2006**	**2006**	EU181200	**genotype III**
**India 07 D1/1CprM/Del07**	**2007**	EU846230	**genotype III**
**India 07 D1/2CprM/Del07**	**2007**	EU846231	**genotype III**

'DENV-1 sequenced in the study are written in bold'

Indian isolates sequenced in this study are in Bold. Each isolate is denoted by country of isolation and last two digits of year of isolation, followed by virus ID.

## Competing interests

The authors declare that they have no competing interests.

## Authors' contributions

HK and PKD contributed equally to this work and carried out the sequencing experiments and phylogenetic analysis and drafted the manuscript. MMP, AC, PS, RSR carried out the collection of samples and carrying out RNA extraction and RT-PCR experiments. MC, DB coordinated with MCD for collection of clinical samples used in this study. SL provided overall scientific and infrastructural support. PVLR provided scientific support and supervision. VM and AR provided overall supervision and critical analysis at every level of scientific work.
